# Lifestyle is associated with thyroid function in subclinical hypothyroidism: a cross-sectional study

**DOI:** 10.1186/s12902-021-00772-z

**Published:** 2021-05-28

**Authors:** Kejun Wu, Yu Zhou, Sujie Ke, Jingze Huang, Xuelin Gao, Beibei Li, Xiaoying Lin, Xiaohong Liu, Xiaoying Liu, Li Ma, Linxi Wang, Li Wu, Lijuan Wu, Chengwen Xie, Junjun Xu, Yanping Wang, Libin Liu

**Affiliations:** 1 Department of Endocrinology and Metabolism, Fujian Medical University Union Hospital, 29 Xinquan Road, Fujian 350001 Fuzhou, China; 2 Department of Clinical Pharmacy and Pharmacy Administration, School of Pharmacy, Fujian Medical University, 350122 Fuzhou, Fujian China

**Keywords:** Subclinical hypothyroidism, Lifestyle, Thyroid function, Cross-sectional study

## Abstract

**Background:**

Few studies have focused on the association between lifestyle and subclinical hypothyroidism (SCH). The purpose of this study was to investigate the association between lifestyle and thyroid function in SCH.

**Methods:**

This study was a part of a community-based and cross-sectional study, the Epidemiological Survey of Thyroid Diseases in Fujian Province, China. A total of 159 participants with SCH (81 males and 78 females) and 159 euthyroid (87 males and 72 females) participants without any missing data were included in the analysis. General information and lifestyle information including sleep, exercise, diet and smoking habits of the participants was collected by questionnaire and Pittsburgh sleep quality index scale (PSQI) was collected. Thyroid stimulating hormone (TSH), free thyroxine (FT4), thyroid peroxidase antibody (TPOAb), thyroid globulin antibody (TgAb) and urine iodine concentration (UIC) were tested. Thyroid homeostasis parameter thyroid’ s secretory capacity (SPINA-GT), Jostel’s TSH index (TSHI), thyrotroph T4 sensitivity index (TTSI) were calculated. Logistic regression and multiple linear regression were performed to assess associations.

**Results:**

Compared with euthyroid subjects, patients with SCH were more likely to have poor overall sleep quality (15.1 vs.25.8 %, *P* = 0.018) and l less likely to stay up late on weekdays (54.7 vs. 23.9 % *P* < 0.001). In SCH group, exercise was the influencing factor of TSH (*β*= -0.224, *P* = 0.004), thyroid secretory capacity (*β* = 0.244, *P* = 0.006) and thyrotropin resistance (*β* = 0.206, *P* = 0.009). Iodine excess was the influencing factor of thyroid secretory capacity (*β* = 0.209, *P* = 0.001) and pituitary thyroid stimulating function (*β* = 0.167, *P* = 0.034). Smoking was the influencing factor of pituitary thyroid stimulating function (*β* = 0.161, *P* = 0.040). Staying up late on weekends was the influencing factor of thyroid secretory capacity (*β* = 0.151, *P =* 0.047). After adjusting for possible confounders, logistic regression showed that those with poor overall sleep quality assessed by PSQI and iodine excess had an increased risk of SCH (OR 2.159, 95 %CI 1.186–3.928, *P* = 0.012 and OR 2.119, 95 %CI 1.008–4.456, *P* = 0.048, respectively).

**Conclusions:**

Lifestyle including sleep, smoking, diet and exercise was closely related to thyroid function especially thyroid homeostasis in SCH.

**Supplementary Information:**

The online version contains supplementary material available at 10.1186/s12902-021-00772-z.

## Background

Subclinical hypothyroidism (SCH) refers to the abnormal thyroid function with increased serum thyrotropin (TSH) level and normal total thyroxine (TT4) and free thyroid hormone (FT4) levels. The prevalence of SCH ranges from 4.6 to 16.7 % around the worldwide [1]. For lacking of obvious clinical manifestations such as fear of cold, constipation, apathy and depression, SCH is mainly found accidentally in routine physical examination and is easy to be ignored by patients. About 5 % of SCH may gradually progress to clinical hypothyroidism without treatment; many studies have suggested that increased TSH is associated with cardiovascular disease risk, insulin resistance, and metabolic syndrome [2, 3]. Therefore, SCH needs to be paid enough attention and corresponding measures should be taken to reduce its harm.

Lifestyle is associated with a variety of endocrine and metabolic diseases, such as obesity, dyslipidemia, metabolic syndrome and type 2 diabetes. Among thyroid diseases, researchers have pay attention to the relationship between lifestyle and SCH. Studies have shown that changes in smoking habits could affect the occurrence of clinical hypothyroidism and SCH [1]; iodine deficiency and iodine excess both increase the incidence of overt hypothyroidism and SCH [4]; compared with sleep duration of 7–8 h, shorter and longer sleep duration was associated with an increased risk of SCH [5]; people who were sedentary had higher levels of TSH than those who exercised regularly [6], and SCH was associated with decreased exercise tolerance [7].However,. there are few studies on how lifestyle affects thyroid function in SCH. Thyroid function is regulated by hypothalamus and pituitary. The changes of thyroid hormone are regulated by the negative feedback of hypothalamus pituitary thyroid (HPT) axis. We speculate that for SCH, the dysfunction of HPT axis may occur before TSH increasing. Basic thyroid functional parameters like TSH and T4 may not reflect the dynamic change of HPT axis accurately. Therefore, novel parameters obtained by mathematical modeling were created as supplementary indicators [8–10]. Thyroid homeostasis parameters reflect the functional status of HPT axis, which can be used as a new perspective to evaluate the impact of lifestyle on thyroid function in patients with SCH.

Therefore, the purpose of this study was to investigate the association between lifestyle and thyroid function in SCH by analyzing the correlation between SCH and lifestyle including sleep quality and habit, exercise, diet and smoking, and the association between lifestyle and thyroid homeostasis in patients with SCH.

## Methods

### Study population

This cross-sectional study was conducted in 2016 in the Fujian Province of China. All the participants enrolled the national epidemiological survey (Tide) aimed to determine the prevalence of thyroid diseases and diabetes and the iodine nutrition status[11]. The subjects were enrolled according to the following criteria: (1) adult residents aged over 18 years; (2) Han race; (3) and living in Fujian for at least 5 years. The exclusion criteria were as follows: (1) receiving iodine contrast agent examination or taking iodine-containing drugs in recent three months; (2) pregnant and breastfeeding women. A total of 2,651 individuals were randomly selected from the general population in urban (*n* = 1394) and rural (*n* = 1257) areas. For further analysis of population with SCH, subjects with elevated serum TSH levels and normal FT4 levels were considered as subclinical hypothyroidism [12]. The following were exclusion criteria: (1) patients with previously diagnosed thyroid diseases, including hyperthyroidism, hypothyroidism, thyroid tumors, thyroid nodules, etc., regardless of whether they have been treated; (2) patients with normal thyroid function, but with morbid syndrome acute and chronic renal insufficiency, adrenocortical hypofunction and other diseases that may cause abnormal increase of TSH. The study was carried in accordance to Declaration of Helsinki. The protocol was approved by the Ethics Committee of Fujian Medical University Union Hospital (Grant no.2015KY032). Subjects with high TSH levels (> 4.2 mIU/L) and normal free T4 (FT4) levels (12.0–22.0 pmol/L) were diagnosed with SCH (*n* = 214). Subjects with a history of thyroid disease treatment, such as surgery, radiation, and thyroid hormone or antithyroid medication (n = 21) and with missing data (n = 34) were further excluded. There were 159 participants with, including 81males and 78 females (mean age = 42.4 ± 15.8 years) in the final analysis. Age and gender matched subjects were selected from normal TSH population participating in this epidemiological investigation as the control group by propensity score matching. The control group consisted in 159 euthyroid (EUTH) subjects, including 87 males and 72 females (mean age = 40.6 ± 15.0 years) without previous history of thyroid disease and treatment and missing data.

### Demographic and anthropometric variables

The general data such as name, sex, age, occupation, education level and past medical history were collected by questionnaire. The data of height, weight, waist circumference (WC), systolic blood pressure (SBP), diastolic blood pressure (DBP) were measured. Height and weight were measured in light clothing without shoes. Waist circumference (WC) was measured using a tape measure placed halfway between the lower border of the ribs and the iliac crest in a horizontal plane. Height, weight and WC were measured twice and the averages were taken. Height, WC and weight were measured to the nearest 0.1 cm and 0.1 kg, respectively. Body mass index (BMI) was calculated by dividing the participant’s weight (kg) by the square of height (m^2^).

### Laboratory measurements

After fasting for 8 h at night, the venous blood samples were collected between 08:00 am to 10:00 am. Thyroid hormones and thyroid autoantibodies, including thyroid stimulating hormone (TSH), free thyroxine (fT4), and free triiodothyronine (fT3) thyroid peroxidase antibody (TPOAb), thyroid globulin antibody (TgAb) were quantified using the immunochemiluminometric assay (ICMA) method, using the standard kits from Roche Diagnostics GmbH (Germany). To screen the prevalence of thyroid disorders in the population, fT4 and fT3 were measured only among those with abnormal levels of TSH. Fasting blood glucose (FBG), serum total cholesterol (TC), triglyceride (TG), high-density lipoprotein cholesterol (HDL-C), low-density lipoprotein cholesterol (LDL-C), and uric acid (UA) were measured by an automated procedure (BS180; Mindray, Ltd., Shenzhen, China). Hemoglobin (Hb) A1c was measured by HPLC using an automated analyzer (V ARIANT™ II TURBO, BioRad, Berkeley, USA). Midstream urine samples were collected in the morning, and urinary iodine concentration (UIC) was quantified using the ammonium persulfate method. According to the standard put forward by WHO/UNICEF/ICCIDD in 2007 [13], UIC > 300 µ g / L is regarded as iodine excess.

### Thyroid homeostasis assessment

Thyroid homeostasis parameter thyroid’ s secretory capacity (SPINA-GT), Jostel’s TSH index (TSHI), thyrotroph T4 sensitivity index (TTSI, also referred to thyrotroph thyroid hormone resistance index or thyrotroph thyroxine resistance index) were calculated.

SPINA-GT provides an estimate of the maximum thyroid secretory rate under stimulation conditions. The maximum stimulating effect of TSH on the thyroid and the dissociation, protein binding, distribution and elimination of FT4 are involved in SPINA-GT. It was sensitive to thyroid diseases of primary origin and specific for secondary dysfunction of thyroid [9]. Previous studies have observed that SPINA-GT correlates with thyroid volume measured by ultrasound, and increases in hyperthyroidism and decreases in hypothyroidism [14]. It was calculated as

$$\widehat{G}=\frac{{\beta }_{T} \left({ D}_{T}+\left[TSH\right] \right) \left(1+{K}_{41}\left[TBG\right]+{K}_{42}\left[TBPA\right]\right) \left[F{T}_{4}\right]}{{\alpha }_{T}\left[TSH\right]}$$

In this formular, TBG referred to standard concentration of thyroxine-binding globulin, and TBPA referred to standard transthyretin concentration. Constants in these formulas were as follows: β_T_ = 1.1 × 10^− 6^/s, D_T_ = 2.75 mU/L, K_41_ = 2.0 × 10^10^ L/mol, TBG = 300 nmol/L, K_42_ = 2.0 × 10^8^ L/mol, TBPA = 4.5 µmol/L and α_T_ = 0.1/L.

The function of the pituitary was evaluated by TSHI. It estimated the maximum pituitary TSH reserve by correcting the negative feedback suppression of TSH by measured FT4 concentration and extrapolating the standardized uninhibited TSH assuming a FT4 value of 0, providing an accurate estimate of the severity of pituitary dysfunction. As a supplementary indicator of pituitary function, it provided a sufficiently accurate and sensitive assessment of the severity of hypopituitary function by basic thyroid function test in the absence of dynamic pituitary function test[15]. Its reduction was thought to be associated with gonadotropin insufficiency, lower peak concentrations of growth hormone and cortisol in pituitary stimulation tests, and it was used in the assessment of non-thyroid disease syndroms (NTIS) [9]. It was calculated as

$$TSHI=\text{ln}\left( \left[TSH\right] \right)+0.1345\times \left[{FT}_{4}\right]$$

An additional index used for assessing thyrotropic function and TSH feedback inhibition was TTSI. It was calculated by the equilibrium concentrations of TSH and free T4 and the upper limit of the reference interval of FT4 (l_u_) [9]. It represented the feedback of the HPT axis, and its elevation was associated with thyroid hormone resistance and pituitary dysregulation. It has been used to evaluate the dysregulation of the HPT axis due to genetic mutations and genetic factors [16, 17]. It was calculated as

$$TTSI=\frac{100\left[TSH\right]\left[F{T}_{4}\right]}{{l}_{u}}$$

### Assessment of lifestyle

Lifestyle data including sleep, diet, smoking and exercise were collected by questionnaire. The Pittsburg sleep quality index scale (PSQI) was used to assess the overall sleep quality of subjects. PSQI is a sleep quality scale compiled by Buysse et al. in 1989 [18] which has a high correlation with the results of polysomnography and is simple and feasible with high reliability and validity. PSQI includes seven dimensions: subjective sleep quality, sleep latency, sleep duration, habitual sleep efficiency, sleep disturbances, use of sleeping medications, and daytime dysfunction. The total score of PSQI ≥ 7 in this study was regarded as poor overall sleep quality. Living habits were collected by questionnaire including: (1) sleep habits: lunch break (more than 30 min or not), bedtime at night on weekdays and weekends (later than 10:00 pm or not), Sleep duration on weekdays and weekends (less than 5 h, 5–6 h, 7–8 h, 9–10 h and more than 10 h); (2) exercise habits: exercise for more than 3 days a week, lasting more than 30 min once (no, low intensity and medium or high intensity); (3) eating habits: daily salt intake (less than 5 g 5 ~ 10 g, more than 10 g), type of salt intake (iodized salt or not), iodine-rich foods intake like kelp and laver (none, monthly and daily). (4) and smoking habit (yes or no).

### Statistical analysis

All the statistical analyses were performed by the SPSS version 26.0 (IBM SPSS Inc.). The continuous variables were reported as mean values and standard deviations or as median values and corresponding 25th and 75th percentiles. The percentages were reported for the categorical variables. Student’s T test or Mann-Whitney U test was used to analyze the differences in continuous variables between SCH group and NC group. Chi-squared test or Fisher’s exact test were performed to assess differences in categorical variables between the two groups. Because TTSI levels were distributed in a skewed manner, they were reciprocally transformed before statistical analysis. Correlation between thyroid homeostasis variables and thyroid homeostasis parameters were calculated using the Pearson or Spearman correlation coefficient. Logistic regression was used to analyze the risk factors of SCH. Age, gender, BMI, SBP, TgAb, TC, UA and HbA1c were included in logistic regression as confounding factors to evaluate whether a risk factor was independently associated with SCH. Multiple linear regression was used to further analyze the influence of lifestyle on thyroid homeostasis parameters in SCH group. Statistically significant factors were selected from correlation analysis as independent variables, and the independent variables were screened by forward method to establish the model. The lifestyle variables with statistically significant (*P* < 0.05) such as exercise, stay up late on weekends, iodine excess and smoking were included in the model, that is, the influencing factors of thyroid homeostasis parameters. *P <* 0.05 was considered to be statistically significant.

## Results

### General characteristics

**Fig. 1:**
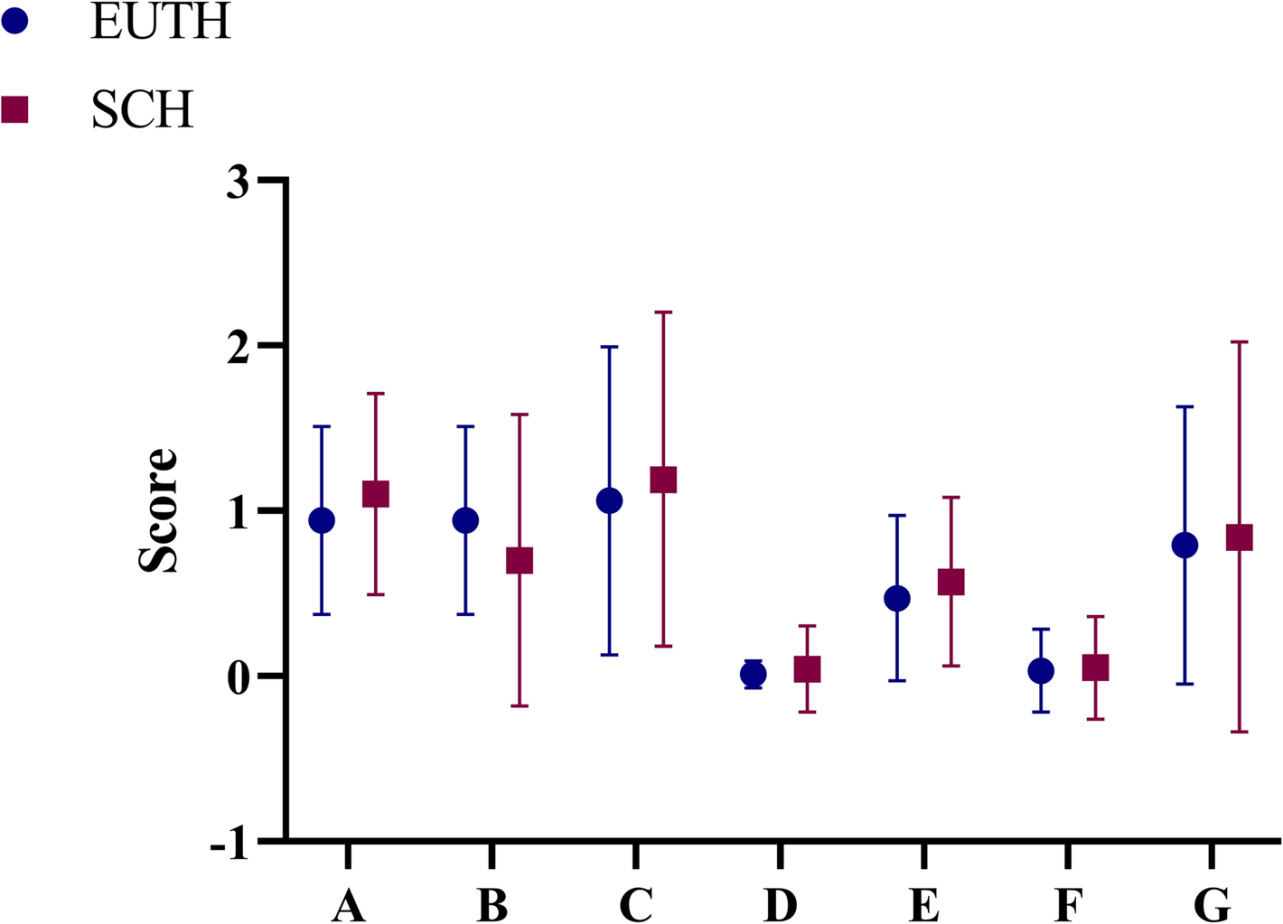
Score of seven dimensions of the Pittsburg sleep quality index scale (PSQI). **A**, subjective sleep quality; **B**, sleep latency; **C**, sleep duration; **D**, habitual sleep efficiency; **E**, sleep disturbances; **F**, use of sleeping medications; **G**, daytime dysfunction

There were 159 participants in EUTH group and SCH group respectively. General characteristics of the population are as shown in Table 1. There were no significant difference in age and gender between EUTH subjects (mean age 40.6 ± 15.0 years, females 45.3 %) and patients with SCH (mean age 42.4 ± 15.8 years, females 49.1 %).Compared with euthyroid subjects, patients with SCH were more likely to have poor overall sleep quality (15.1 vs.25.8 % *P* = 0.018) and less likely to stay up late on weekdays (54.7 vs. 23.9 % *P* < 0.001). There was significant different in sleep duration on weekends categorized into less than 5 h, 5 ~ 6 h, 7 ~ 8 h, 9 ~ 10 h and more than 10 h between EUTH subjects and patients with SCH (1.3, 15.1, 59.1, 24.5, 0 % vs. 1.9, 20.8, 61.6, 11.9, 3.8 %, *P* < 0.001). TSH, positive rate of TPOAb and TgAb were significantly increased in patients with SCH compared with EUTH subjects (5.01 mIU/L vs.1.92 mIU/L *P* < 0.001, 19.5 % vs. 8.8 % *P* = 0.006 and 26.4 % vs. 6.3 % *P <* 0.001 respectively). There was no significant difference in lunch break, stay up late on weekends, sleep duration on weekdays, exercise, dietary habits related to iodine intake, UIC and smoking between SCH group and NC group (*P >* 0.05). There was also a trend towards higher BMI (23.9 ± 3.2 kg/m^2^ vs. 22.6 ± 3.1 kg/m^2^, *P <* 0.001), WC (81.7 ± 9.5 cm vs. 77.8 ± 10.6 cm, *P* = 0.001), SBP (127.0 ± 19.0 mmHg vs. 122.3 ± 16.2 mmHg, *P* = 0.019) and DBP (80.0 ± 11.6 mmHg vs. 76.4 ± 10.8mmHg, *P* = 0.013), in patients with SCH compared with EUTH subjects. The subjects in SCH group had higher FBG, HbA1c, TG, TC, LDL-C, UA and lower HDL-C than that in EUTH group although there was no significant difference between the two groups. Comparison of sleep quality between EUTH group and SCH group are as shown in Fig. 1. The total scores of PSQI of patients with SCH were significantly higher than those of EUTH subjects (4.50 ± 2.83 vs. 3.88 ± 2.29, *P* = 0.034). Among the seven dimensions of PSQI, the scores of subjective sleep quality of patients with SCH were significantly higher than those of EUTH subjects (1.10 ± 0.61 vs. 0.94 ± 0.57, *P* = 0.018), and the scores of sleep latency of patients with SCH were significantly higher than those of EUTH subjects (0.70 ± 0.88 vs. 0.94 ± 0.57, *P* = 0.003). There was no significant difference in sleep duration, habitual sleep efficiency, sleep disturbances, sleep medication use, and daytime dysfunction between the two groups.

**Table 1:** Characteristics of subjects with and without subclinical hypothyroidism

Characteristics	EUTH (n = 159)	SCH (n = 159)	*P value
Female, %	45.3	49.1	0.500
Age, years	40.6 ± 15.0	42.4 ± 15.8	0.297
**Anthropometric variables**			
BMI, kg/m^2^	22.6 ± 3.1	23.9 ± 3.2	**< 0.001**
WC, cm	77.8 ± 10.6	81.7 ± 9.5	**0.001**
SBP, mmHg	122.3 ± 16.2	127.0 ± 19.0	**0.019**
DBP, mmHg	76.4 ± 10.8	80.0 ± 11.6	**0.013**
**Laboratory measurements**			
TSH, mIU/L	1.92 (1.42, 2.51)	5.01 (4.60,6.13)	**< 0.001**
TPOAb +, %	8.8	19.5	**0.006**
TgAb +, %	6.3	26.4	**< 0.001**
UIC, µg/L	140.70 (89.28, 222.40)	152.20 (99.73,227.90)	0.275
FBG, mmol/L	5.04 (4.69, 5.66)	5.17 (4.80, 5.50)	0.262
HbA1c, %	5.8 ± 0.9	5.9 ± 1.0	0.472
TG, mmol/L	0.98 (0.69, 1.37)	1.08 (0.81, 1.59)	0.102
TC, mmol/L	4.88 ± 1.09	5.09 ± 1.19	0.087
HDL-C, mmol/L	1.37 ± 0.40	1.35 ± 0.38	0.151
LDL-C, mmol/L	2.44 ± 0.70	2.57 ± 0.79	0.712
UA, mmol/L	340.35 ± 95.15	346.59 ± 92.61	0.554
**Lifestyle information**			
PSQI ≥ 7, %	15.1	25.8	**0.018**
More than 30 min lunch break, %	44.7	52.8	0.145
Stay up late on weekdays, %	54.7	23.9	**< 0.001**
Stay up late on weekends, %	67.3	62.9	0.410
Sleep duration on weekdays (< 5 h/5 ~ 6 h/7 ~ 8 h/9 ~ 10 h/>10 h), %	1.9/27.7/57.9/12.6/0	4.4/32.7/52.2/8.2/2.5	0.087
Sleep duration on weekends (< 5 h/5 ~ 6 h/7 ~ 8 h/9 ~ 10 h/>10 h), %	1.3/15.1/59.1/24.5/0	1.9/20.8/61.6/11.9/3.8	**0.003**
Exercise and intensity (no/low/medium or high), %	38.4/44.0/17.6	42.1/35.2/14.5	0.242
Daily salt intake (< 5 g/5 ~ 10 g/>10 g), %	14.5/73/12.6	14.5/73/12.6	0.759
Iodized salt intake, %	95.0	89.3	0.061
Iodine-rich foods intake (none/monthly/daily), %	11.9/70.4/17.6	13.2/71.1/15.7	0.872
Smoking, %	18.9	16.4	0.556

*P value < 0.05 was considered significant

Data are shown as mean (SD), median (interquartile range), or percentage

*PSQI* Pittsburg sleep quality index scale; *UIC* urinary iodine concentration; *TSH* thyroid stimulating hormone; *TPOAb* thyroid peroxidase antibody; *TgAb* thyroid globulin antibody; *BMI* body mass index; *WC* waist circumference; *SBP* systolic blood pressure; *DBP* diastolic blood pressure; *FBG* fasting blood glucose; *HbA1c* glycosylation hemoglobin; *TG* triglyceride; *TC* total cholesterol; *HDL-C* high density lipoprotein cholesterol; *LDL-C* low density lipoprotein cholesterol; *UA* uric acid

### Association between lifestyle and thyroid function in SCH

Spearman correlation analysis showed that in SCH group, TSH was negatively correlated with exercise (*r=* -0.309, *P* < 0.001), SPINA-GT was positively correlated with stay up late on weekends (*r* = 0.163, *P =* 0.040), exercise (*r* = 0.197, *P =* 0.013), smoking (*r* = 0.196, *P =* 0.013) and iodine excess (*r* = 0.216, *P =* 0.006), and negatively correlated with eating iodine-rich foods intake (*r*=-0.164, *P =* 0.039); TSHI was positively correlated with smoking (*r* = 0.166, *P =* 0.036) and iodine excess (*r* = 0.191, *P =* 0.016); and TTSI^− 1^ was positively correlated with exercise (*r* = 0.223, *P =* 0.005) *P<*(Table 2).Multiple linear regression analysis showed that in the SCH group, the independent variables iodine excess (*β* = 0.209, *P =* 0.001), exercise(*β* = 0.244, *P =* 0.006), and stay up late on weekends (*β* = 0.151, *P =* 0.047) were included in the model of SPINA-GT. The independent variables smoking (*β* = 0.161, *P* = 0.040) and iodine excess (*β* = 0.167, *P* = 0.034) were included in the model of TSHI. The regression type of TTSI^− 1^ was statistically significant, and The independent variable exercise (*β*=-0.224, *P* = 0.004 and *β* = 0.206, *P* = 0.009, respectively) was included in the model of TSH and TTSI^− 1^. All the models above were statistically significant (all *P* < 0.05).It was suggested that the effects of the above variables on TSH and thyroid homeostasis parameters were statistically significant *P*<**(**Table 3**)**.

**Table 2:** Correlation between lifestyle and thyroid homeostasis in subclinical hypothyroidism

Parameters	TSH		SPINA-GT		TSHI		TTSI^− 1^	
	*r*	*P* value	*r*	*P* value	*r*	*P* value	*r*	*P* value
PSQI score	**-**0.039	0.626	0.050	0.533	-0.017	0.830	0.043	0.588
More than 30 min lunch break	-0.062	0.440	0.094	0.238	0.020	0.798	0.017	0.832
Stay up late on weekdays	-0.099	0.213	0.124	0.120	0.042	0.060	0.006	0.939
Stay up late on weekends	-0.148	0.063	0.163	**0.040**	0.052	0.513	0.014	0.865
Sleep duration on weekdays	0.133	0.095	-0.071	0.373	0.124	0.120	-0.150	0.058
Sleep duration on weekends	0.097	0.221	-0.006	0.941	0.094	0.240	-0.084	0.293
Exercise	-0.309	**< 0.001**	0.197	**0.013**	-0.141	0.077	0.223	**0.005**
Daily salt intake	0.051	0.525	-0.125	0.118	-0.070	0.382	0.033	0.683
Iodized salt intake	-0.063	0.434	-0.067	0.402	-0.144	0.071	0.125	0.113
Iodine-rich foods intake	0.149	0.061	-0.164	**0.039**	-0.028	0.725	-0.025	0.753
Iodine excess	-0.020	0.806	0.216	**0.006**	0.191	**0.016**	-0.125	0.117
Smoking	-0.039	0.625	0.196	**0.013**	0.166	**0.036**	-0.104	0.193

*P value < 0.05 was considered significant

*PSQI* Pittsburg sleep quality index scale

**Table 3:** Multiple linear regression of lifestyle and thyroid homeostasis in subclinical hypothyroidism

Dependent variable	Influence factors	*β*	*P* value
TSH	Exercise	-0.224	**0.004**
SPINA-GT	Iodine excess	0.209	**0.001**
	Exercise	0.244	**0.006**
	Stay up late on weekends	0.151	**0.047**
TSHI	Smoking	0.161	**0.040**
	Iodine excess	0.167	**0.034**
TTSI^− 1^	Exercise	0.206	**0.009**

*P value < 0.05 was considered significant

*SPINA-GT* secretory capacity of the thyroid gland; *TSHI* Jostel’s TSH index; *TTSI* thyrotroph thyroid hormone resistance index

### Lifestyle factors associated with SCH

**Fig. 2:**
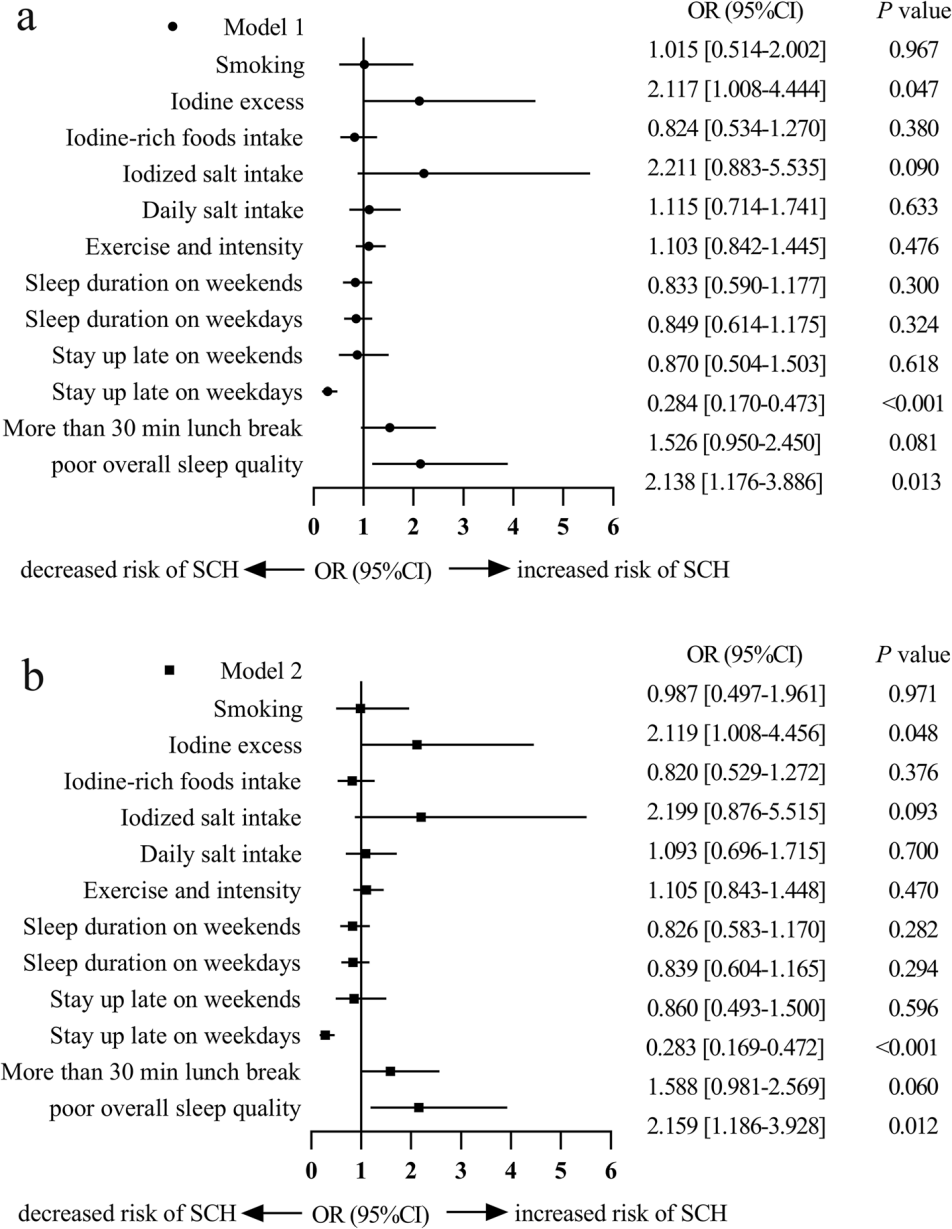
The risk factor and protection factor of SCH adjusted by possible confounding factors. Model 1 adjusted age, sex, BMI, SBP, TgAb. Model 2 adjusted age, sex, BMI, SBP, TgAb. TC, UA, HbA1c. The risk of SCH in subjects with poor overall sleep quality was higher than that with good overall sleep quality. The risk of SCH in subjects with iodine excess was higher than that without iodine excess. And the risk of SCH in those who stayed up late on weekdays was lower than who slept early on weekdays (*P* < 0.05)

To identify lifestyle factors associated with SCH, we analyzed age, sex, physical indicators, thyroid autoantibody and lifestyle factors in a logistic regression model. For the collinearity existed between BMI and WC, SBP and DBP, and TPOAb and TgAb, BMI, SBP and TgAb were selected to represent obesity index, blood pressure level and thyroid autoimmunity respectively. In model 1 adjusted age, sex, BMI, SBP and TgAb, logistic regression showed that those with poor overall sleep quality assessed by PSQI had an increased risk of SCH (OR 2.138, 95 %CI 1.176–3.886, *P* = 0.013), and those who slept late on weekdays had a lower risk of SCH (OR 0.284, 95 %CI 0.170–0.473, *P* < 0.001), and those who with iodine excess had a higher risk of SCH (OR 2.117, 95 %CI 1.008–4.444, *P* = 0.047). In order to further adjust the effect of metabolic factors, TC, UA and HbA1c were added in model 2, which represented blood lipid, blood uric acid and blood glucose respectively. Logistic regression showed the associations above between lifestyle factors with SCH remained the same (poor overall sleep quality OR 2.159, 95 %CI 1.186–3.928, *P* = 0.012, stay up late on weekdays OR 0.283, 95 %CI 0.169–0.472, *P* < 0.001 and iodine excess OR 2.119, 95 %CI 1.008–4.456, *P* = 0.048, respectively). Lunch break, sleep duration, exercise and smoking etc. were not correlated with SCH under either model 1 or model 2 (Fig. 2).

## Discussion

In this study, a cross-sectional study based on community population was conducted to analyze the association between lifestyle and thyroid function of SCH patients from the point of view of HPT axis function and thyroid homeostasis. It was suggested by the results that lifestyle including sleep, diet, exercise and smoking was closely related to the thyroid function of SCH.

The present study suggests that compared with the euthyroid subjects, SCH patients had poorer overall and subjective sleep quality, shorter sleep latency and lower proportion of late sleep on weekdays. The sleep duration of SCH patients was different from that of EUTH group. However, there was no difference in exercise, diet and smoking habits between SCH group and EUTH group in this study. The relationship between lifestyle and SCH has been concerned by some researchers.

Previous studies have shown that lack of sleep can affect the function of the HPT axis [19]. TSH is the main hormone which affected by circadian rhythm in the HPT axis. TSH is secreted instantly during 20:00 to 03:00, and reaches a peak at about 02:00 to 03:00, and then decreases gradually [20]. The relationship between SCH and sleep quality can be explained by the inhibitory effect of sleep on TSH secretion. Lack of sleep is associated with a nearly two-fold increase in the level of TSH at night, suggesting that adequate and high-quality sleep during night can inhibit TSH levels, while compensatory sleep during daytime did not suppress TSH levels below the daytime levels. The mechanism may be that the HPT axis under the control of the circadian clock via the suprachiasmatic nucleus pacemaker, and compensatory sleep after sleep deprivation violates the original circadian rhythm, resulting in the secretion of TSH cannot follow the circadian cycle fluctuations[21]. However, prolonged sleep can also affect the TSH secretion, which may be related to circadian rhythm disorders. A cross-sectional study suggests that shorter and longer sleep periods are associated with subclinical thyroid dysfunction compared with the optimal sleep duration 7-8 h per night [5]. The relationship between HPT axis function and sleep may not be one-way, and some studies have shown that the dysfunction of HPT axis can lead to a decline in sleep quality [22]. The hypothalamic suprachiasmatic nucleus is the control center of circadian rhythm [21]. It is reported that there are two forms of TSH, free TSH and macro TSH, in human body. Free TSH is synthesized in thyrotrophs of the pars distalis (PD) of the pituitary gland and stimulates synthesis and release of thyroxine. Macro TSH is secreted or complex-formed at the pars tuberalis (PT) adjacent to the pituitary and is controlled by suprachiasmatic nucleus which regulates circadian rhythms [23]. The increase of serum macro TSH is related to poor sleep quality [24]. In order to evaluate the relationship between the function of HPT axis and sleep quality, thyroid homeostasis parameters such as SPINA-GT, reflecting the maximum secretory capacity of the thyroid gland [9], and TSHI and TTSI, reflecting the thyroid secretory function of the pituitary gland [10] were introduced in this study. Univariate analysis and multiple linear regression suggested that staying up late on weekends was a factor affecting thyroid secretory function in patients with SCH. However, the *P* values (0.040 and 0.047, respectively) were close to the critical value of significance test, suggesting that further study is needed to explore the association between staying up late on weekends and thyroid secretion function.

To the best of our knowledge, there is no consensus on the correlation between thyroid function and exercise so far. Studies have shown that exercise training can improve the quality of life, sit and reach test results and carotid intima-media thickness and reduce body fat ratio in women with SCH, but has no significant effect on thyroid-related hormones [25, 26]. In this study, although there was no significant difference, the proportion of people with regular physical activity in control group was higher than that of SCH group. Patients with hypothyroidism are known to have reduced excitability and inactivity due to thyroid hormone deficiency, but whether inactivity occurs in patients with SCH without an overt decrease in thyroid hormone has not been discussed in detail. The present study suggested that in SCH group, exercise intensity was negatively correlated with TSH and positively correlated with SPINA-GT and TTSI^− 1^. Multiple linear regression showed that exercise was included in the regression model of TSH, SPINA-GT and TTSI^− 1^ respectively, suggesting that regular, moderate and high intensity exercise is related to lower TSH, better thyroid secretion and lower thyrotropin resistance. Although the present study does not yet clarify whether patients with SCH show a state of inactivity before the thyroid hormone is significantly decreased, it shows the correlation between SCH and inactivity, as well as the correlation between moderate and high intensity exercise and better thyroid function in patients with SCH.

Studies have shown that iodine deficiency [27] and iodine excess [28] are risk factors for hypothyroidism and SCH. This study suggested that in SCH group, SPINA-GT and TSHI were positively correlated with iodine excess. And in multiple linear regression, iodine excess was included in the regression model of SPINA-GT and TSHI respectively, suggesting that iodine excess can enhance thyroid secretion and the ability of the pituitary gland to promote thyroid secretion. Studies on rats have shown that the iodization of thyroid hormone in thyroid was inhibited when iodine was excessive, and the synthesis of thyroxine was decreased, which contributed to the increased secretion of TSH by pituitary and release of thyroid hormones that have been synthesized in the thyroid gland. As a result, the serum thyroxine level was normal while the serum TSH level was increased [29], showing the performance of SCH. In physiological conditions, pituitary secretion of TSH promotes thyroid secretion of thyroxine, and when serum thyroxine increases, it produces negative feedback regulation on pituitary to reduce TSH secretion. It is precisely because of this negative feedback regulation that TSH and serum thyroxine levels can not accurately reflect the ability of pituitary to promote thyroid secretion and thyroid secretion[30]. It is necessary to be evaluated by thyroid homeostasis parameters. In some individuals, iodine excess can induce hypothyroidism, although thyroid function may recover after iodine withdrawal[31]. In the SCH group, we observed that there was a negative correlation between SPINA-GT and iodine-rich foods intake, but there was no correlation between iodine excess and iodine-rich foods intake, the possible reason was that iodine nutrition status is related to dietary iodine intake, while the frequency and amount of iodine-rich foods intake, the storage mode of iodine-rich food and the cooking method of iodine-rich foods can affect the intake of iodine [32]. Therefore, the relationship between SPINA-GT and iodine nutrition status should be evaluated by UIC rather than the frequency of iodine-rich foods intake.

Studies have shown that among women with SCH, the average TSH level of smokers is higher than that of non-smokers, and the clinical performance score of smokers shows that their hypothyroidism is more severe [33]. Some studies also suggested that the prevalence of elevated serum TSH was significantly decreased in smokers [34], and the risk of autoimmune-related hypothyroidism increased significantly after quitting smoking[35], suggesting that smoking may be a protective factor for hypothyroidism. This study showed that SPINA-GT and TSHI were positively correlated with smoking in SCH group, suggesting that smoking was related to better thyroid secretion and pituitary thyroid-promoting function. Multiple linear regression suggested that smoking was included in the regression model of TSHI, suggesting that smoking is one of the influencing factors of pituitary thyroid-promoting function.

The main cause of SCH is thyroid autoimmunity, and studies have found that SCH is associated with dyslipidemia, insulin resistance and renal dysfunction [36]. It was found in this study that the positive rate of thyroid antibody in SCH group was significantly higher than that in EUTH group. Although there was no significant difference, SCH group had higher FBG, HbA1c, TG, TC, LDL-C, UA and lower HDL-C than EUTH group. In order to clarify whether the relationship between lifestyle and SCH was affected by these factors, we analyzed the lifestyle factors that may affect SCH by logistic regression.

There were some limitations in the present study. First, the study was a cross-sectional study, which can not clarify the causal relationship between lifestyle and SCH. Second, the subjected included in this study was small and all Han ethnicity from Fujian province, China with overall adequate iodine nutrition, which may be accompanied by selection bias. Therefore, large, prospective multicenter studies in different regions, nations and races should be conducted, and the different iodine nutrition statues should be considered.

## Conclusions

In this study, a community-based cross-sectional study was conducted to analyze the relationship between lifestyle and thyroid function in SCH. It was found that lifestyle including sleep, smoking, diet and exercise was closely related to SCH. Staying up late on weekends, exercise, iodine excess and smoking were the influencing factors of thyroid function in SCH.

## Supplementary Information

**Additional file 1.**

## Data Availability

The data used to support the findings of this study are available from the corresponding author upon reasonable request.

## Abbreviations

SCH: Subclinical hypothyroidism
TSH: Thyroid stimulating hormone
FT4: Free thyroxine
HPT: Hypothalamus- Pituitary- Thyroid
TPOAb: Thyroid Peroxidase Antibody
TgAb: Thyroglobulin Antibody
BMI: Body mass index
WC: Waist circumference
WHtR: Waist height ratio
SPINA-GT: Thyroid’ s secretory capacity
TSHI: Jostel’ s TSH index
TTSI: Thyrotroph thyroid hormone resistance index
SBP: Systolic blood pressure
DBP: Diastolic blood pressure
FBG: Fasting blood glucose
HbA1c: Glycosylation hemoglobin
TG: Triglyceride
TC: Total cholesterol
LDL-C: Low density lipoprotein cholesterol
HDL-C: High density lipoprotein cholesterol
PSQI: Pittsburg sleep quality index scale
UIC: Urinary iodine
