# Supplementary material for: Lifestyle is associated with thyroid function in subclinical hypothyroidism: a cross-sectional study
Source: BMC Endocr Disord. 2021 May 28;21:112. doi: 10.1186/s12902-021-00772-z (PMC8161919; doi:10.1186/s12902-021-00772-z)
Supplement: Supplementary file 1 — Additional file 1. [file 12902_2021_772_MOESM1_ESM.docx]

**Lifestyle is associated with thyroid function in subclinical hypothyroidism:** **a cross-sectional study**

Kejun Wu^1#^, Yu Zhou^1, 2#^, Sujie Ke^1^, Jingze Huang^1^, Xuelin Gao^1^, Beibei Li^1^,

Xiaoying Lin^1^, Xiaohong Liu^1^, Xiaoying Liu^1^, Li Ma^1^, Linxi Wang^1^, Li Wu^1^,

Lijuan Wu^1^, Chengwen Xie^1^, Junjun Xu^1^, Yanping Wang^1*^, Libin Liu^1*^

^1^ Department of Endocrinology and Metabolism, Fujian Medical University Union Hospital, Fuzhou 350001, Fujian, China.
^2^ Department of Clinical Pharmacy and Pharmacy Administration, School of Pharmacy, Fujian Medical University, Fuzhou 350122, Fujian, China

^#^ Kejun Wu and Yu Zhou contributed equally to this work.

^*^ **Corresponding Author**:

Libin, Liu and Yanping Wang

Department of Endocrinology and Metabolism, Fujian Medical University Union Hospital, 29 Xinquan Road, Fuzhou 350001 Fujian, China

E-mail: libinliu@fjmu.edu.cn (Libin Liu)

yp1014wang@163.com (Yanping Wang)

Supplementary material

Questionnaire of Lifestyle

*Thank you for taking the national epidemiological survey (Tide) aimed to determine the prevalence of thyroid diseases and diabetes and the iodine nutrition status. The Questionnaire of Lifestyle may take you eight minutes or so to complete. Please fill in the form according to your situation. Thank you very much for your kind support.*

No. __________________ Date: _______________

**Personal information**

1. Name: __________________

2. Sex: 1) male 2) female

3. Date of birth: _____________

4. Province: ________________

5. Location: ________________

6. Nationality: 1) Han 2) Zang 3) Uyghur 4) Hui 5) Zhuang 6) others

**Iodine intake**

1. Source of edible salt: 1) commercially iodized salt 2) non-iodized salt

2. Salt intake habit: 1) severe (> 10 g/person/day)

2) moderate (5-10 g/person/day)

3) mild (less than 5 g/person/day)

*Reference quantity: toothpaste cap =5 g beer cap =10 g*

3. Have you eaten kelp or nori for three days? 1) yes 2) no

4. Seaweed intake :1) often (weekly) 2) occasionally (monthly) 3) no

5. Currently taking iodine-containing drugs: 1) yes 2) no

6. If yes, what drug 1) amiodarone 2) potassium iodide 3) kelp

4) Traditional Chinese medicine (TCM) containing iodine

5) iodine vitamin 6) others

7. Have you taken a contrast examination in the last three months? 1) yes 2) no

**Smoking history**

1. Do you smoke? 1) no 2) yes

**History of thyroid disease**

1. Does anyone in your family have thyroid disease? 1) yes 2) no

If yes, the type of disease is:

1) hyperthyroidism 2) hypothyroidism 3) goiter 4) thyroid nodule 5) thyroid cancer

6) thyroid adenoma 7) others

2. Have you ever suffered from thyroid disease? There is no 2= no

If yes, the type of disease is

1) hyperthyroidism 2) hypothyroidism 3) goiter 4) thyroid nodule 5) thyroid cancer

6) thyroid adenoma 7) others

**Exercise habits**

1. Do you exercise regularly (at least 30 minutes each time, 3 days a week or more)?

1) no

2) low intensity (walking, gymnastics, etc.)

3) medium intensity (brisk walking, jogging, table tennis, Tai Chi, square dancing, etc.)

4) high intensity (basketball, swimming, badminton, running, mountain climbing, etc.)

**Pittsburgh Sleep Quality Index (PSQI)**

*The following questions relate to your usual sleep habits during the past month only. Your answers should indicate the most accurate reply for the majority of days and nights in the past month. Please answer all questions.*

1. During the past month, what time have you usually gone to bed at night?

BED TIME __________(weekday) _________(weekend)

2. During the past month, how long (in minutes) has it usually taken you to fall asleep each night?

NUMBER OF MINUTES __________(weekday) _________(weekend)

3. During the past month, what time have you usually gotten up in the morning?

GETTING UP TIME _________(weekday) __________(weekend)

4. During the past month, how many hours of ç)) did you get at night? (This may be different than the number of hours you spent in bed.)

HOURS OF SLEEP PER NIGHT __________(weekday) __________(weekend)

For each of the remaining questions, check the one best response. Please answer all questions.

5. During the past month, how often have you had trouble sleeping because you...

a) Cannot get to sleep within 30 minutes

1) Not during the past month 2) Less than once a week

3) Once or twice a week 4) Three or more times a week

b) Wake up in the middle of the night or early morning

1) Not during the past month 2) Less than once a week

3) Once or twice a week 4) Three or more times a week

c) Have to get up to use the bathroom

1) Not during the past month 2) Less than once a week

3) Once or twice a week 4) Three or more times a week

d) Cannot breathe comfortably

1) Not during the past month 2) Less than once a week

3) Once or twice a week 4) Three or more times a week

e) Cough or snore loudly

1) Not during the past month 2) Less than once a week

3) Once or twice a week 4) Three or more times a week

f) Feel too cold

1) Not during the past month 2) Less than once a week

3) Once or twice a week 4) Three or more times a week

g) Feel too hot

1) Not during the past month 2) Less than once a week

3) Once or twice a week 4) Three or more times a week

h) Had bad dreams

1) Not during the past month 2) Less than once a week

3) Once or twice a week 4) Three or more times a week

i) Have pain

1) Not during the past month 2) Less than once a week

3) Once or twice a week 4) Three or more times a week

j) Other reason(s), please describe_________________________________________

How often during the past month have you had trouble sleeping because of this?

1) Not during the past month 2) Less than once a week

3) Once or twice a week 4) Three or more times a week

6. During the past month, how would you rate your sleep quality overall?

1) Very good

2) Fairly good

3) Fairly bad

7. During the past month, how often have you taken medicine to help you sleep (prescribed or over the counter)?

1) Not during the past month 2) Less than once a week

3) Once or twice a week 4) Three or more times a week

8. During the past month, how often have you had trouble staying awake while driving, eating meals, or engaging in social activity?

1) Not during the past month 2) Less than once a week

3) Once or twice a week 4) Three or more times a week

9. During the past month, how much of a problem has it been for you to keep up enough enthusiasm to get things done?

1) No problem at all

2) Only a very slight problem

3) Somewhat of a problem

4) A very big problem

10. Do you have a bed partner or roommate?

1) No bed partner or room mate

2) Partner/roommate in other room

3) Partner in same room, but not same bed

4) Partner in same bed

If you have a roommate or bed partner, ask him/tier how often in the past month you have had...

a) Loud snoring

1) Not during the past month 2) Less than once a week

3) Once or twice a week 4) Three or more times a week

b) Long pauses between breaths while asleep

1) Not during the past month 2) Less than once a week

3) Once or twice a week 4) Three or more times a week

c) Legs twitching or jerking while you sleep

1) Not during the past month 2) Less than once a week

3) Once or twice a week 4) Three or more times a week

*Thank you again for your kind support!*

All the participants of this cross-sectional study enrolled the national epidemiological survey (Tide) aimed to determine the prevalence of thyroid diseases and diabetes and the iodine nutrition status[1]. The relevant content in the questionnaire is from the Tide study. In addition, the authors collected additional data on exercise and sleep. The PSQI was designed by Buysse et al. [2] to evaluate overall sleep quality in clinical populations.

Reference

1. Li Y, Teng D, Ba J, Chen B, Du J, He L, Lai X, Teng X, Shi X, Li Y *et al*: **Efficacy and Safety of Long-Term Universal Salt Iodization on Thyroid Disorders: Epidemiological Evidence from 31 Provinces of Mainland China**. *Thyroid* 2020, **30**(4):568-579.

2. Buysse DJ, Reynolds CF, 3rd, Monk TH, Berman SR, Kupfer DJ: **The Pittsburgh Sleep Quality Index: a new instrument for psychiatric practice and research**. *Psychiatry Res* 1989, **28**(2):193-213.
